# Alkaline hydrogen peroxide pretreatment of cladodes of cactus (opuntia ficus-indica) for biogas production

**DOI:** 10.1016/j.heliyon.2021.e08002

**Published:** 2021-09-16

**Authors:** Jemal Beshir Belay, Nigus Gabbiye Habtu, Venkata Ramayya Ancha, Ali Seid Hussen

**Affiliations:** a Department of Sustainable Energy Engineering, Jimma Institute of Technology, Jimma University, Jimma, Ethiopia; b Department of Chemical Engineering, Bahir Dar Institute of Technology, Bahir Dar University, Bahir Dar, Ethiopia; c Department of Thermal Engineering, Jimma Institute of Technology, Jimma University, Jimma, Ethiopia

**Keywords:** Cactus, Alkaline hydrogen peroxide pretreatment, Delignification, Anaerobic digestion

## Abstract

The alkaline hydrogen peroxide (AHP) pretreatment of cladodes of cactus (Opuntia ficus-indica) for biogas production was evaluated based on the delignification of cladodes of cactus. The effects of alkaline hydrogen peroxide concentration (30% w/w solution) and the pretreatment time (3, 6, 9, and 12 h) were evaluated at pH 11.5, temperature of 30 °C, and 180 rpm for removal of lignin.

A batch of anaerobic digestion experiments were conducted at mesophilic temperature conditions (37 ± 1 °C) with the pretreated biomass. The feed stock (cladodes of cactus) used in this study contained 12.51 ± 1.25 cellulose, 16.34 ± 2.93% hemicellulose, and 10.45 ± 2.31% lignin, and the balance were (carbohydrate, protein, lipid, and ash). After AHP pretreatment, the lignocellulosic content of the feed stock was changed to 12.50 ± 1.84%, 13.63 ± 3.23%, and 7.49 ± 3.05% for cellulose, hemicellulose, and lignin respectively. The AHP pretreatment of cladodes of cactus highly affected the lignin structure relative to cellulose and hemicellulose. The alkaline hydrogen peroxide pretreatment resulted in a higher amount of biogas produced from 877.9 ± 15.12 ml biogas/g VS to 1613.5 ± 10.76 ml biogas/g VS which is an 83.4% increment and decreased after 9 h treatment to 1398.8 ± 17.8 ml biogas/g VS. In addition, the measured methane yields range from 302.48 ± 0.33 to 602.65 ± 3.24 ml CH_4_/g VS. The results showed that alkaline hydrogen peroxide pretreatment of cladodes of cactus is an effective strategy for enhance biogas yield.

## Highlights

• Biogas production from alkaline hydrogen peroxide (AHP) pretreated cladodes of cactus was evaluated.
• The effect of alkaline hydrogen peroxide (AHP) pretreatment on the lignocellulosic structure of cladodes of cactus was analyzed by scanning electron microscope (SEM).
• Alkaline hydrogen peroxide (AHP) pretreatment improves lignin disintegration by 28.89%.
• AHP pretreatment improves the production of biogas by 83.4%.

## 1 Introduction

In recent years, bioenergy research has become a focus of many research tailored to the pretreatment of lignocellulose biomass to improve the energy yield. Among the bio-energies sources, biogas production from biomass has been recognized worldwide as a low-cost and easily accessible energy source for low-income countries.

It yields also a good quality of organic fertilizer. In addition, it helps to reduce environmental pollution. Biogas is a gas fuel that is derived from the decay of organic matter, as a mixture of mainly methane (CH_4_) and carbon dioxide (CO_2_). It also consists of trace amount of other components, including hydrogen sulfide (H_2_S), nitrogen oxide (NO_X_), and ammonia (NH_3_), and other volatile compounds. Biogas can be produced from energy crops, agricultural waste, and various residential and industrial waste materials. However, energy crops need fertile land to grow that is used for agricultural activities, while residential and industrial wastes need different treatment mechanisms to clean them from different unwanted substances that inhibit microbial action in the biogas production routes and the biomethane potential is low [1]. Those feedstocks (energy crops, and various residential and industrial waste materials) are less attractive for commercial biogas production due to their dependence on the sources and need lengthy treatment process. Due to this, current research is focused on the improvement of biogas yield produced from pretreating lignocellulosic feed stocks and producing biogas from CAM (Crassulacean acid metabolism) plants such as cactus (OFI) biomass. Nowadays, biogas production from CAM plants such as cactus is a promising due to its biogas potential, availability and environmental advantages [2, 3, 4, 5, 6, 7]. According to P. S. Calabrò et.al. [4], pretreatment of cactus for biogas production improves both the quantity and quality of biogas. G. Antonopoulou and G. Lyberatos [8], reported that the pretreatment of lignocellulosic feedstock improves the biological degradation of the substrate. According to M. J. Taherzadeh and K. Karimi [9], the pretreatment method can enhance hydrolysis and avoid the creation of inhibitory by-products. There are different pretreatment techniques such as physical, physico-chemical, chemical, and biological treatments that help in increasing the biological degradation [10]. The pretreatment method can enhance hydrolysis and avoid the generation of inhibitory by-products [9].

According to P. S. Calabrò et.al. [4], alkaline pretreatment has a double effect on the lignocellulosic content of the cladodes of cactus, that was de-crystalline the cellulose and reduce lignin content. The same researcher reported a methane yield of cladodes of cactus (OFI) ranged from 420 to 600 NmL/g VS after acidic pretreatment. According to M. K. Gray [11], hydrogen peroxide pretreatment (AHP) is a good choice for lignocellulosic feedstocks becouse it gives a high yield of glucose at moderate temperature and pressure, However, information on the effect of alkaline hydrogen peroxide (AHP) on cladodes of cactus is lucking. This study focuses mainly on exploring the impact of AHP pretreatment on cladodes of cactus (opuntia ficus-indica) for the production of biogas. The main advantage of Cladodes of cactus (OFI) is; (1) it does not compete with the land used for agricultural activities, (2) it can tolerate cold and dry weather conditions unlike the conventional crops [5]. In addition, the cultivation of cactus (OFI) helps to improve soil regeneration to protect the soil from erosion by creating a vegetative cover in dry regions of the world [12].

## 2 Materials and methods

### 2.1 Raw material and chemicals

The cladodes of cactus (Opuntia) used as a substrate for anaerobic digestion was obtained from Adama City, Oromia, Ethiopia. The cultivated area is located at 8°32′00″ N 39°14′46″ E. It was a greenish, pad-like section of the cactus (Opuntia ficus-indica) and was collected from uncultivated land. Cladodes of cactus (OFI) were chopped by hand with a knife (12–14 cm) and washed with water, sliced using a knife and crushed by using a juicer (Geepas, GSB5362), oven-dry (intercontinental equipment, DAS42000) at 105 °C for 24 h, crushed (mortar and pestle), and passed through a mesh with a size of 20–80 mesh (0.25–0.84 mm). The milled cladodes of cactus were stored at 4 °C.

Sodium hydroxide (98%, Tianjin Cheng yuan Chemical Co., Ltd, China), hydrogen peroxide (30%w/w, Xing Tai Xingjian new material technology co., ltd. Hebei, China), sulphuric acid (98%, Brenntag, France), potassium dichromate (99.8%, MSCA, Germany), orthophosphoric acid (>85%, Foodchem International Corporation, China), ferrous ammonium sulfate (>98%, HPYCHEMICALS, Germany) and copper sulfate (99.9%, Tianjin Cheng yuan Chemical Co., Ltd, China) were the major chemicals used for this study.

### 2.2 Determination of moisture content, total solid and volatile solid

The moisture content was determined according to standard methods [13], about 10 g of fresh sample was taken and weighed and placed in a clean, dry dish. The measured sample was placed in the oven at 105 °C for 24 h then removed, cooled in a desiccator, and weighed. Total solids (TS) and volatile solids (VS) of the substrate were examined by the oven drying and furnace ignition methods [13]. For total solid determination, clean empty porcelain crucibles were heated at 550 °C for about 1 h and cooled in a desiccator at room temperature. The empty crucibles were weighed and a fresh 10 g of sample was added. It was then oven-dried (intercontinental equipment, DAS42000) for 24 h at 105 °C. After which the crucibles with the content were cooled in the desiccator and their weight was recorded. For volatile solid determination, the same samples were further heated in a furnace (BDX-Resistance furnace, SX 2.5–12) at 550 °C for 3 h. The samples were cooled in the desiccator, weighed and calculated the volatile solid and total solid.

### 2.3 Determination of total carbon, total nitrogen, cellulose, hemicellulose and lignin

Total carbon determination was done by using the modified Walkley-Black method [14]. According to EPA [15], 0.1 g of dry samples was put in 250 ml conical flasks and 10 ml of 1N K_2_Cr_2_O_7_ was added and swirled. Then 15 ml of H_2_SO_4_ was added in a fume hood, swirled again three times, and the flasks were stood for 30 min and 150 ml of distilled water was added, followed by the addition of 5 ml of Orthophosphoric acid and the remaining dichromate was examined by redox titration with 0.5 N ferrous ammonium sulfate (Fe (NH_4_)_2_(SO_4_)_2_) till the total solution color change from blue to green. A blank was run without a sample. The nitrogen was determined by the Kjeldahl method [13], the total nitrogen content, which involved the sample digestion and volumetric determination whereby about 1 g of sample with a catalyst (5 g of K_2_SO_4_ and 0.5 g of CuSO_4_ and 15 ml of concentrated H_2_SO_4_) was weighed and added into a digestion flask. Until the digestate color changed to blue, the mixtures were heated. Subsequently, the digestate was cooled and transferred into a 100 ml volumetric flask. Blank digestion was also made. 10 ml of diluted digestate was transferred into a distilling flask and 15 ml of 40% NaOH was added and washed with about 2 ml of distilled water. Then 60 ml of digestate was distilled and the distillate was titrated using 0.02N HCl until an orange color was observed. Calculations were done according to the formula.

(1) $$Total\;Nitrogen=\;\left(V_{1}-V_{2}\right)\times N\times f\times 0.014\times \frac{100}{V}\times \frac{100}{S}$$

where, V_1_ = Titer for the sample (ml); V_2_ = Titer for blank (ml), N = Normality of standard HCl solution, f = Factor of standard HCl solution, V = Volume used for distillation, and S = Weight of sample taken (g).

To determine cellulose, hemicelluloses, and lignin, a direct method was used [16]. For this about 2 g of fiber was boiled in ethanol for 15 min (four times), washed carefully with distilled water, and kept in an oven at 40 °C for 24 h. Then divided into two parts, A and B. The fraction B was treated with 24% KOH for 4 h at 25 °C, washed carefully with distilled water, and dried at 80 °C for 24 h and weighted. The same sample was treated with 72% H_2_SO_4_ for 3 h to hydrolyze the cellulose and refluxed with 5% H_2_SO_4_ for 2 h and washed carefully with distilled water and dried at 80 °C for 24 h and the dry weight was taken as the C fraction. The biomass composition was calculated as

(2) Cellulose = B - C, Hemicellulose = A - B and Lignin = C itself [16]

### 2.4 Determination of feed stock morphology

The morphology of the untreated cladodes of cactus and alkaline hydrogen peroxide (AHP) pretreated cladodes of cactus was determined using scanning electron microscopy (SEM) (a JCM-6000Plus microscope). 15 KV was the operating voltage.

### 2.5 Effect of alkaline hydrogen peroxide pre-treatment on methane yield

To determine the effect of alkaline hydrogen peroxide pretreatment on cladodes of cactus for biogas production, experiments were carried out at four different contact time of alkaline hydrogen peroxide (AHP) exposure: 3, 6, 9, and 12 h. For each AHP pretreatment experimental run, 10 g of milled cladodes of cactus was added to a 250 ml Erlenmeyer flask and added a 50 ml prepared alkaline solution (NaOH solution at pH 11.5) and soaked for 5 min. It was measured by using a general electrode probe (biogas 5000, geotechnical instrument, UK, Ltd) and adjusted pH to 11.5 through the addition of small aliquots of NaOH. An H_2_O_2_ loading of 0.125 g H_2_O_2_/g biomass is desired for the effective delignification of biomass [17, 18, 19]. A 30% (w/w) stock solution of H_2_O_2_ was used for this pretreatment. After the addition of peroxide, the pH was adjusted to a pH of 11.5 by using NaOH. A Teflon thin films were used to seal the top of each flask to avoid evaporation and volume expansion of the mixture due to oxygen evolution during pretreatment without changing the pretreatment conditions (pressure). Finally, the flasks were placed in an incubator with orbital shaking (Excella E24R, New Brunswick Scientific Edison, New Jersey, USA) set at 30 °C; with a mixing rate of 180 rpm. After 3, 6, 9, and 12 h of total mixing time, the flasks were removed from the shaker and the pH was adjusted to a range of 6.7 using 72% (w/w) H_2_SO_4_ and the samples were prepared for anaerobic digestion.

### 2.6 Batch anaerobic digestion

A mixture of three cladodes of cactus samples was prepared and used for the AHP pretreatment anaerobic digestion process. The mixture was prepared by mixing a total of 50 g from three samples of cladodes of cactus and that is used for 5 treatments (untreated, 3,6,9 and 12 h AHP pretreatment) 10 g of sample for each treatment. The source of inoculum used to feed the system was active slurry. In this study, 500 ml Erlenmeyer flasks were prepared for lab scale anaerobic digestion. The flasks were connected to a gas tight plastic airbag with gas tight plastic tubes for biogas collection and a rubber stopper for taking biogas samples. Anaerobic digestion was conducted in an optimum mesophilic condition (37 ± 1 °C). The bio reactor was organized into three batches (sets). The first set included three digesters that were charged with untreated cladodes of cactus substrate for the conventional method of digestion and three digesters that were charged with 3 h alkaline hydrogen peroxide treated cladodes of cactus. The second set consisted of two groups of bioreactors with 6 and 9 h alkaline hydrogen peroxide treated cladodes of cactus. The third set consisted of three bioreactors with 12 h alkaline hydrogen peroxide treated cladodes of cactus. A biogas and methane measurement were done by using a gas syringe and gas analyzer (biogas 5000, geotechnical instruments (UK). ltd) until no significant amount of biogas was produced (for 30 days).

### 2.7 Data analysis

Each experiment was conducted in triplicate and the data was expressed as mean ± standard error (SE). T-tests as well as correlation statistics at 5% significant level (p = 0.05), respectively, were carried out using IBM SPSS Statistics 20 software.

## 3 Results and discussion

### 3.1 Substrate characterization

#### 3.1.1 Feed stock (cladodes of cactus)

The cactus (Opuntia Ficus-Indica) used in this study was found growing randomly dispersed intermittently. There were relatively short, almost invisible spines on the cladodes. Cladodes of cactus characteristics are useful in distinguishing species given that cladode size has been suggested to be species dependent [20]. In this study, the cladode has a dimension of 11–20 cm width and 28–35 cm length (Table 1). This is same range of the reported values of Opuntia Ficus Indica, 12–29 cm, and 24–67 cm for cladode width and height respectively [21] and [2]. The greenish color of the cladode suggests that the opuntia samples analyzed in this study were young (1–2 years) [2, 22], and [23]. It has been reported that the physicochemical composition of opuntia can vary depending on the species of the plant and the climate of where they were grown [24].

**Table 1:** Morphological characteristics of cladodes of cactus from collected samples.

Sample	Cladode color	Spin	Cladode Weight (kg)	Cladode Length (cm)	Cladode Width (cm)
1	Green	Absent	5.2	31	18
2	Green	Absent	3.8	28	11
3	Green	Absent	4.5	35	20

Note; The collected sample of cladodes of cactus were selected by its size from small to big.

#### 3.1.2 Moisture content, total solid and volatile solid

The feedstock moisture content (% MC), total solid (% TS), and volatile solid (% VS) values were stated as mean ± standard error (SE) (Table 2). The mean moisture content of cladodes of cactus was found to be 94.7 ± 0.057%. It is noted that the moisture content of cladodes of cactus found in this study is relatively higher than other studies. And the mean value of total solid was 5.3 ± 0.057% which is lower than other researchers' results found in the range of 8–14% [3, 5, 7], and [2]. According to S. S. Sadaka and C. R. Engler [25] as the moisture content of the feedstock increases, the bacteria can access easily the liquid substrate and that increases digestion. This could be due to the result of higher moisture content of the cladodes of the cactus.

**Table 2:** Characteristics of feed stock (% moisture content, %TS and %VS) T = test.

Parametres	T1 (%)	T2 (%)	T3 (%)	Mean	Standard Error (SE)
Moisture content	94.6	94.7	94.8	94.7	0.05774
TS	5.4	5.3	5.2	5.3	0.05774
VS as percentage of TS	76.5	78	71.9	75.47	1.83515

In addition, the content of total solids and moisture content depended on the growing area of the cactus plant and the type of species. In this study, the mean value of volatile solid (VS) was 75.47 ± 1.835% that is in agreement with the works of several authors results 78.2%, 78%, 74.5% and 80%. [3, 4, 7], and [26]. The result showed that the large fraction of cladodes of cactus were biodegradable and they were important for biogas production.

#### 3.1.3 Total carbon, total nitrogen, cellulose, hemicellulose, and lignin

The mean total carbon content was 33.5 ± 0.03% and the total nitrogen was 1.2 ± 0.02% with a corresponding carbon and nitrogen ratio (C/N) of 27.9. The C/N ratio obtained in this study is within the recommended range (20–30) for biogas generating microbial consortiums [27]. The AHP pretreated cladodes of cactus used in this study contained 12.51 ± 1.25 to 12.50 ± 1.84% cellulose, 16.34 ± 2.93% to 13.63 ± 3.23% hemicellulose, and 10.45 ± 2.31% to 7.43 ± 3.05% lignin (Table 3). The amount of lignin was close to those reported in the literature, such as 11.02 ± 0.61% by M. Hawa et.al [2]. and higher than the values of 7.7% ± 0.41% and 11% by P. S. Calabrò [4]. According to A. J. B. Zehnder and W. Gujer [28], lignocellulose biomass is resistant to hydrolysis due to its structural lignin barrier, so it is necessary to pretreat the biomass to improve the hydrolysis stage. In this study, the mean lignin content decreased from 10.45 ± 2.31% to 7.49 ± 3.05%. This indicated that the lignin barrier was minimized that may improve hydrolysis process during the anaerobic digestion. P. F. de Souza Filho et.al. [5], reported that minimizing the lignin content increases the convergence of polysaccharides into fermentable sugars that improves the anaerobic digestion process. The lignin value of Opuntia reported by different researchers was between 3.32 to 8.3% [2, 4, 29, 30]. The higher values of lignin (8.6% ± 0.43%) reported in Tanzania [2], and the lower values of lignin (3.32%) reported in Italy [4] (see Tables 4, 5, 6, 7).

**Table 3:** Cellulose, hemicellulose and lignin content of feedstock.

	Untreated OFI	3 h AHP treated	6 h AHP treated	9 h AHP treated	12 h AHP treated
Cellulose	12.51 ± 1.25	12.50 ± 1.84	12.51 ± 1.54	12.51 ± 1.44	12.50 ± 2.0
Hemicellulose	16.34 ± 2.93	15.71 ± 2.03	14.21 ± 3.63	13.63 ± 3.23	14.13 ± 1.73
Lignin	10.45 ± 2.31	9.35 ± 2.75	8.33 ± 2.15	7.43 ± 3.05	8.21 ± 2.14

**Table 4:** One sample statistic for Moisture, TS and VS content of OFI.

	N	Mean	Std. Deviation	Std. Error Mean
Moisture	3	94.7000	.10000	.05774
TS	3	5.3000	.10000	.05774
VS	3	75.4667	3.17857	1.83515

**Table 5:** T-Test for volume of biogas in each test (untreated OFI, 3, 6, 9 and 12 h AHP treated OFI).

	N	Mean	Std. Deviation	Std. Error Mean
C	3	878.5905	26.19180	15.12184
T1	3	1295.9849	16.91741	9.76727
T2	3	1614.4009	18.63774	10.76050
T3	3	1400.0599	30.84251	17.80693
T4	3	1571.0989	33.38015	19.27204

**Table 6:** T-Test for volume of methane in each test (untreated OFI, 3, 6, 9 and 12 h AHP treated OFI).

	N	Mean	Std. Deviation	Std. Error Mean
C	3	302.1667	.57735	.33333
T1	3	480.0000	7.76209	4.48144
T2	3	586.5000	10.00000	5.77350
T3	3	602.6667	5.61991	3.24465
T4	3	592.5667	8.65814	4.99878

**Table 7:** T-Test for methane percentage in each test (untreated OFI, 3, 6, 9 and 12 h AHP treated OFI).

	N	Mean	Std. Deviation	Std. Error Mean
C	3	45.0889	3.37595	1.94911
T1	3	48.7056	1.30196	.75169
T2	3	47.8500	1.21347	.70059
T3	3	57.5556	4.22484	2.43921
T4	3	48.7944	1.06149	.61285

These values indicated that the lignocellulosic fraction of the cladodes of cactus was climate dependent. From this study the value of lignin was closer to the values that were studied in Tanzania [2].

### 3.2 Scanning electron microscopy (SEM) analysis

**Figure 1:**
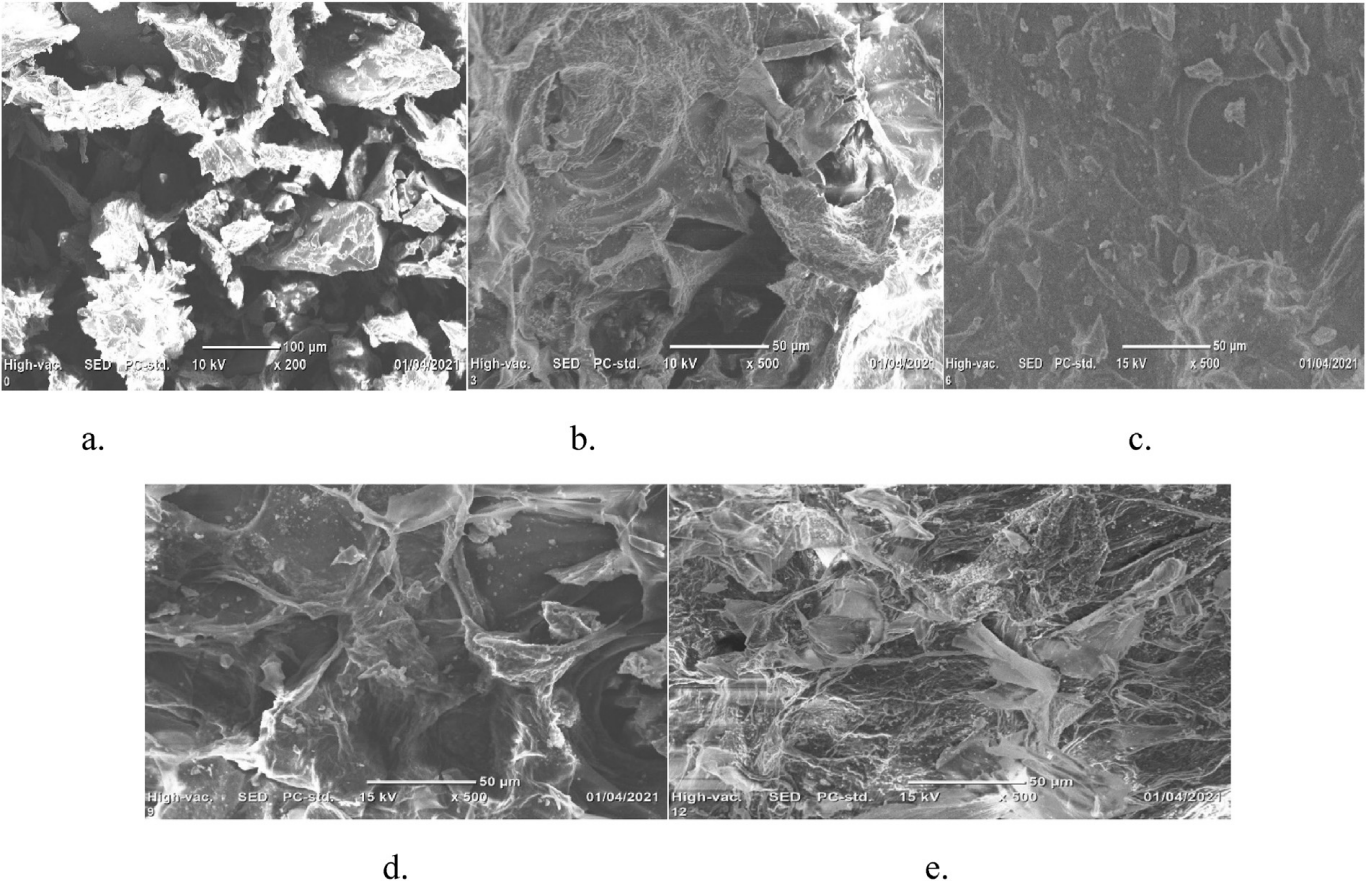
SEM images of the untreated and pretreated OFI biomass. (a) untreated OFI biomass, (b) 3 h AHP treated OFI (C) 6 h AHP treated OFI, (d) 9 h AHP treated OFI and (e) 12 h AHP treated OFI.

AHP pretreated and untreated cladodes of cactus were visualized by using a scanning electron microscope (SEM) to analyze the structural changes (Figure 1). The AHP pretreated cladodes of cactus sample showed the collapse and cracks of the cell wall of the plant, this is due to the degradation of lignin inter units’ linkages [31]. Figure 1 (b, c, d, and e) showed that the cracks and holes of cladodes of cactus. On the other hand, untreated cladodes of the cactus showed a flat and intact surface (Figure 1, a). The SEM result revealed that AHP treatment destabilizes the surface structure of the plant. According to E. C. D. Carvalho et.al. [32], AHP pretreatment disrupt lignin structure, so it helps anaerobic digestion bacteria to access the cellulose component easily for biogas production. The SEM result confirmed the structural degradation of lignin by AHP pretreatment in agreement with the previous studies [4].

### 3.3 Anaerobic digestion of cladodes of cactus

The content of total solids and volatile solids of the inoculum used in the anaerobic digestion were 23.1 ± 0.52% and 91.8 ± 1.97% and the substrate were 5.3 ± 0.06% and 75.47 ± 1.84%. These values were used to calculate the loading rate of anaerobic digester. The total amount of substrate loaded into the 250 ml working volume anaerobic reactor was found to be 10 g. The temperature was adjusted at 37 ± 1 °C and the pH of each digester was also adjusted to 6.7 at the beginning of the digestion process. The pH of AHP treated cladodes of cactus decreased after the digestion process due to the formation of acid by acidogenic bacteria. The mean pH value was 5.6, 5.9, 6.1, 5.9, and 6.0, for untreated, 3 h, 6 h, 9 h, and 12 h AHP pretreated cladodes of the cactus digester, respectively.

### 3.4 Effect of AHP pretreatment on total biogas production

**Figure 2:**
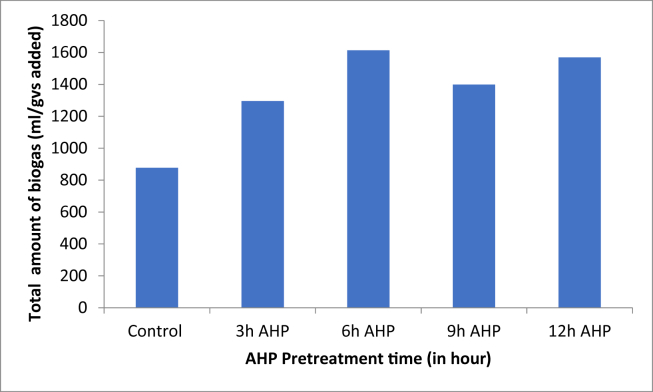
Amount of biogas in each AHP pretreatment time.

**Figure 3:**
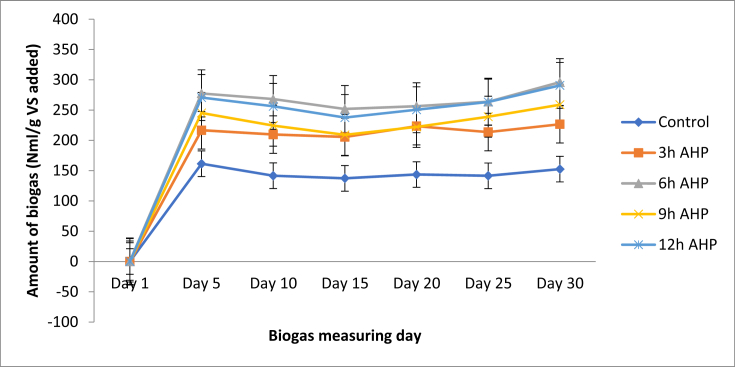
Effect of AHP pretreatment on biogas.

The total biogas production for control (untreated), 3 h, 6 h, 9 h, and 12 h treated samples were 877.9 ± 15.12, 1295.5 ± 9.77, 1613.5 ± 10.76, 1398.8 ± 17.80, and 1569.2 ± 19.27 in ml/g VS respectively. The maximum total biogas production was observed with 6 h pretreatment samples (1613.5 ± 10.76 ml/g VS), followed by a 12 h pretreatment period (1569.2 ± 19.27 ml/g VS) and the least was the control (877.9 ± 15.12 ml/g VS) (Figure 2). As the pretreatment time increased from untreated to 6 h, biogas production had increased from 877.9 ± 15.12 ml/g VS to 1613.5 ± 10.76 ml/g VS. This is an 83.4% increment compared to the untreated samples. This indicates that AHP pretreatment improved the digestibility of the holocellulose component of the substrate and effectively reduced the lignin content (Figure 1). The slight decrease of biogas yield at higher pretreatment time might be due to degradation of the soluble sugars by the alkaline pretreatment [4]. M. J. Taherzadeh and K. Karimi [9], and A. W. T. Owolabi, G [33], reported that approximately 50% delignification was achieved by alkaline treatment (pH of 11.5). According to G. Banerjee et.al. [17], AHP pretreatment time increases enzyme digestibility. In this study, the AHP pretreatment time increases from 3 to 9 h, the yield of biogas increased. After 9 h the biogas production starts decreasing. To select the a best pretreatment time, it is necessary to determine the methane potential of the feedstock to estimate the extent to which the specific feedstock can be degraded [34]. The biogas potential test analysis was measured every 5 days for 30 days and the average measured biogas production for untreated, 3, 6, 9 and 12 h were presented in Figure 3. After the first day of measurement, the production of biogas started to fluctuate and eventually reached close to zero after the 30^th^ day of the retention time. It is also possible that there was an accumulation of toxic substances due to the increased microbial population in the digester, which might have inhibited the microbial action [35]. Soluble biodegradable organic substances are consumed throughout the anaerobic incubation period. Therefore, at the end of the experiment, biogas production declined due to the depletion of those substrates [2].

### 3.5 Biogas quality (% methane)

**Figure 4:**
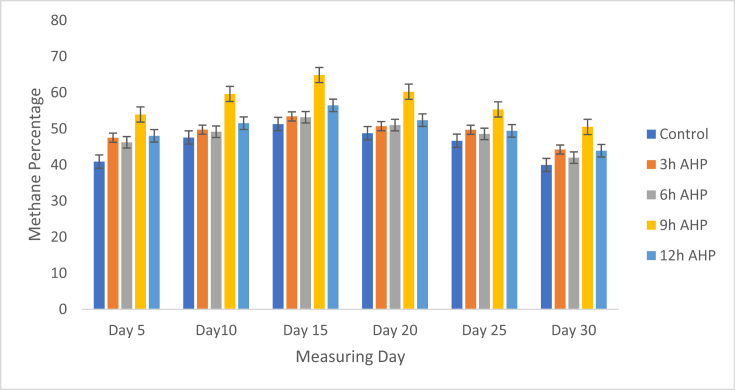
Quality of biogas.

**Figure 5:**
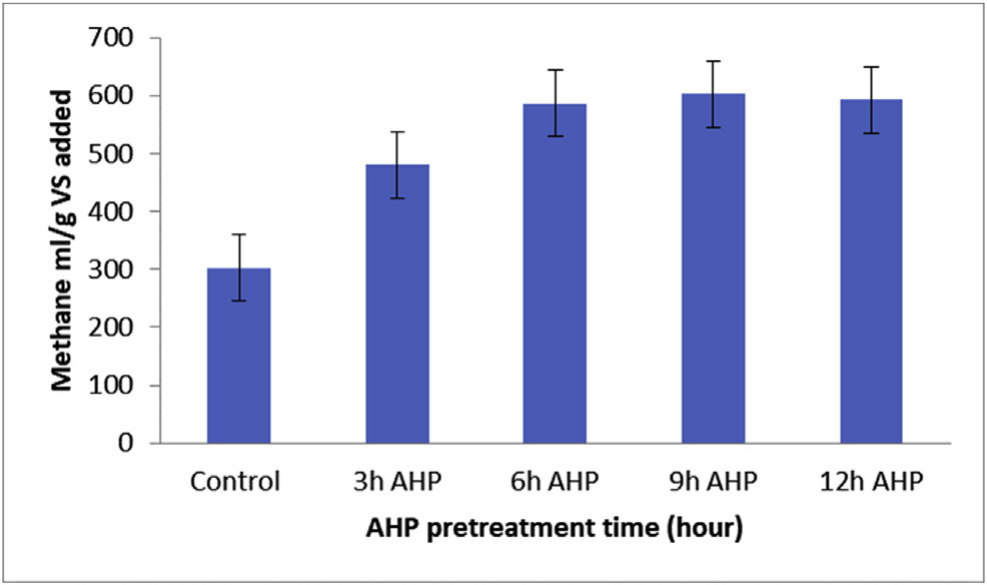
Total amount methane produced in each AHP pretreatment.

The production of biogas and the methane percentage were measured in 5-day intervals. It was found that 9 h pretreatments samples have the highest methane percentage relative to other pretreated samples. At this pretreatment time (9 h AHP pretreatment), 602.65 ± 3.24 mlCH_4_/g VS) of methane gas was measured in 30 days of digestion period. The quality of methane was determined by the percentage of methane produced. In this study, the highest methane percentage was recorded at day 15 of time on stream for 9 h AHP pretreatment samples (64.86% CH_4_) (Figure 4). The measured values of total methane yield (milliliters of methane per gram of volatile solid added) of AHP pretreated cladodes of cactus were presented in (Figure 5). The methane yield increases with pretreatment time up to 9 h and starts decreasing after 12 h AHP pretreatment time. The methane yield varied with feedstock pretreatment time ranging from 480.47 ± 4.45 to 602.65 ± 3.24 mlCH_4_/g VS, whereas that of control was 302.48 ± 0.33 mlCH_4_/g VS. There were limited research findings on the pre-treatment of cladodes of cactus before anaerobic digestion. However, some researchers have reported an increase in biogas and methane percentage due to the pretreatment method [4]. Slight differences in various studies have been reported when working with thermal, alkaline, and acidic pretreatment of cladodes of cactus samples where the highest methane yield was obtained at 20% HCl dosage [4]. The same authors reported that methane yields ranged from approximately 420 NmlCH_4_/g VS for the raw substrate to up to approximately 600 NmlCH_4_/g VS. According to M. Hawa et.al. [2], aerobic pretreatment of cladodes of cactus resulted a value of 0.286 m^3^CH_4_/kg VS to 0.702 m^3^CH_4_/kg VS. The different results could be the use of different pretreatment types, feed composition, and place of plantation. According to C. Sambusiti et.al [36]. AHP pretreatment alters the lignin structure, which improves the biodegradability of the feedstocks. As a result of an increase in methanogenic activity the biogas production increased. In this study, the methane yield decreased after 9 h pretreatment (602.65–592.54 mlCH_4_/g VS) which corresponded to a potential decrease of 1.76%. This shows that further exposure to AHP doesn't have a further benefit, but rather a negative impact on methane yield. A reduction of methane yield in biodigesters with longer hour AHP pretreatment is reported elsewhere [37]. Based on this study findings, the best pretreatment time can be said to lie in the period between 6 to 12 h and further treatment affects the cellulose degradation that generates inhibitors and it undesirably affect the biogas production. This study showed that AHP pretreatment of cladodes of cactus for biogas production was advantageous in enhancing both the biogas production and the percentage of methane yield (biogas quality).

## 4 Conclusions

Anaerobic digestion of cladodes of cactus with and without AHP was performed in a batch anaerobic reactor to assess the biogas production potential and methane yield of cladodes of cactus. The results revealed that the pretreatment of cladodes of cactus with AHP decreased the lignin content and made the structure looser for easy accessibilities of the cellulose part to the microbial action. Consequently, the amount of biogas production was significantly improved from 877.9 ± 15.12 to 1569.2 ± 19.27 ml biogas/g VS. This leads to a maximum methane yield of 602.65 ± 3.24 ml CH_4_/g VS at a 9 h pretreatment time, temperature of 30 °C and 0.125% g/g H_2_O_2_ at an optimal pH of 11.5. The AHP pretreatment shows a promising result for improvement of biogas production from cactus. Nevertheless, further analysis of such pretreatment method at different parameters and treatment conditions is required for possible scale up to the pilot scale experiments.

## Declarations

### Author contribution statement

Jemal Beshir Belay: Conceived and designed the experiments; Performed the experiments; Analyzed and interpreted the data; Contributed reagents, materials, analysis tools or data; Wrote the paper.

Nigus Gabbiye Habtu: Conceived and designed the experiments; Analyzed and interpreted the data; Wrote the paper.

Venkata Ramayya Ancha: Conceived and designed the experiments; Contributed reagents, materials, analysis tools or data.

Ali Seid Hussen: Performed the experiments; Contributed reagents, materials, analysis tools or data.

### Funding statement

This work was supported by Wolkite University, and 10.13039/501100005068Jimma University.

### Data availability statement

Data included in article/supp. material/referenced in article.

### Declaration of interests statement

The authors declare no conflict of interest.

### Additional information

No additional information is available for this paper.
